# Prospective pre- and post-race evaluation of biochemical, electrophysiologic, and echocardiographic indices in 30 racing thoroughbred horses that received furosemide

**DOI:** 10.1186/s12917-018-1336-0

**Published:** 2018-01-18

**Authors:** Catherine T. Gunther-Harrington, Rick Arthur, Krista Estell, Beatriz Martinez Lopez, Alexandra Sinnott, Eric Ontiveros, Anita Varga, Joshua A. Stern

**Affiliations:** 1 Department of Medicine & Epidemiology, School of Veterinary Medicine, University of California Davis, Davis, CA 95616 USA; 2 School of Veterinary Medicine, University of California Davis, Davis, CA 95616 USA; 3 William R. Pritchard Veterinary Medical Teaching Hospital (VMTH), University of California Davis, Davis, CA 95616 USA; 4 Center for Animal Disease Modeling And Surveillance (CADMS), Department of Medicine & Epidemiology, School of Veterinary Medicine, University of California Davis, Davis, CA 95616 USA; 5 Present Address: Gold Coast Veterinary Service & Consulting, Esparto, CA 95627 USA; 6 Present Address: Marion duPont Scott Equine Medical Center, Virginia-Maryland College of Veterinary Medicine, Leesburg, VA 20176 USA

**Keywords:** Troponin, Cardiology, Sudden death, Cardiac ultrasound, Exercise

## Abstract

**Background:**

Exercise induced cardiac fatigue (EICF) and cardiac dysrhythmias are well described conditions identified in high-level human athletes that increase in frequency with intensity and duration of exercise. Identification of these conditions requires an understanding of normal pre- and post-race cardiac assessment values. The objectives of this study were to (1) characterize selected indices of cardiac function, electrophysiologic parameters, and biochemical markers of heart dysfunction prior to and immediately after high level racing in Thoroughbred horses receiving furosemide; and (2) create pre- and post-race reference values in order to make recommendations on possible screening practices for this population in the future.

**Results:**

Thirty Thoroughbred horses were enrolled in the study with an age range of 3–6 years. All horses received furosemide prior to racing. Physical exams, ECGs, and echocardiograms were performed prior to racing (T0) and within 30–60 min following the race (T1). Blood samples were obtained at T0, T1, 4 h post-race (T4) and 24 h after the race (T24). Electrolytes, hematocrit, cardiac troponin I, and partial pressure CO2 values were obtained at all time points. Heart rate was significantly increased post-race compared to baseline value with a median difference of 49 bpm, 95% CI [31,58],(*P* < 0.0001). No dysrhythmias were noted during ECG assessment. Following the race, an increase in number of horses demonstrating regurgitation through the aorta and AV valves was noted. Systolic function measured by fractional shortening increased significantly with a mean difference of 7.9%, 95% CI [4.8, 10.9], (*P* < 0.0001). Cardiac troponin I was not different at pre- and immediately post-race time points, but was significantly increased at T4 (*P* < 0.001). Troponin returned to baseline value by T24.

**Conclusions:**

This study utilized a before and after study design where each horse served as its own control, as such the possible effect of regression to the mean cannot be ruled out. The reference intervals generated in this study may be used to identify selected echocardiographic and electrocardiographic abnormalities in racing horses receiving furosemide.

## Background

Exercise induced cardiac fatigue (EICF) and cardiac dysrhythmias are well described conditions identified in high-level human athletes that increase in frequency with intensity and duration of exercise [1–3]. Characterization of cardiovascular abnormalities including EICF and dysrhythmias in equine athletes are limited, however changes consistent with EICF and significant cardiac arrhythmias have been identified in endurance horses [4–6]. Although racing Thoroughbred horses are not subjected to long duration of exercise, the intensity is unmatched in the performance horse industry. For this reason, there is increased attention to cardiac assessment in race horses [7–14].

EICF is identified by echocardiographic evidence of ventricular systolic and/or diastolic dysfunction. Additionally, post-exercise cardiac arrhythmias, changes to electrophysiologic intervals such as the QT interval and cardiac biomarker elevation are all identified to varying degrees in athletes with EICF [1, 2, 6]. The occurrence of EICF has not been evaluated in Thoroughbred racehorses. To date, there has not been a comprehensive assessment of pre- and post-race parameters in Thoroughbred racing horses, therefore there is a need to establish normal reference intervals so outliers may be identified.

Unexplained sudden cardiac death is of great interest for the race horse industry. Thoroughbred racehorse cases of sudden death after racing have increased, particularly in California over the past 5 years.^1^ Arrhythmic or cardiac causes of these incidences is speculated and warrants further attention [7, 15]. Understanding the normal electrophysiologic, echocardiographic and biochemical values in this population is important if any meaningful cardiac screening practices are to be proposed.

The objectives of this study were to (1) characterize selected indices of cardiac function, electrophysiologic parameters, and biochemical markers of heart dysfunction prior to and immediately after high level racing in Thoroughbred horses receiving furosemide; and (2) create pre- and post-race reference values in order to make recommendations on the best screening practices for race horses.

## Methods

### Animals

Thirty-eight Thoroughbred racehorses were recruited for participation in the study during 2 different race weekends at the Santa Anita Park race track between 2/19/2015 and 3/8/2015. Horses were selected based on availability and owner consent. Consent for participation was obtained from all horse owners. This study was performed in accordance with the institutional animal care and use committee at the University of California Davis (IACUC # 18285). All race horses enrolled had a cardiovascular physical examination performed by one investigator (JAS) and were deemed healthy for racing by the attending track veterinarians in accordance with the racing guidelines and practices. Thorough evaluation of the cardiovascular system and any abnormal heart sounds were recorded [16]. Sex, age, race length (in furlongs), race substrate (dirt or turf), and place at finish were recorded. Horses that were claimed and transferred to a new owner and trainer following the race were excluded as no post-race data could be obtained. All horses were noted to receive furosemide after T0 and prior to T1 in keeping with standard pre-race practices in this racing jurisdiction.

### Electrocardiography

**Fig. 1:**
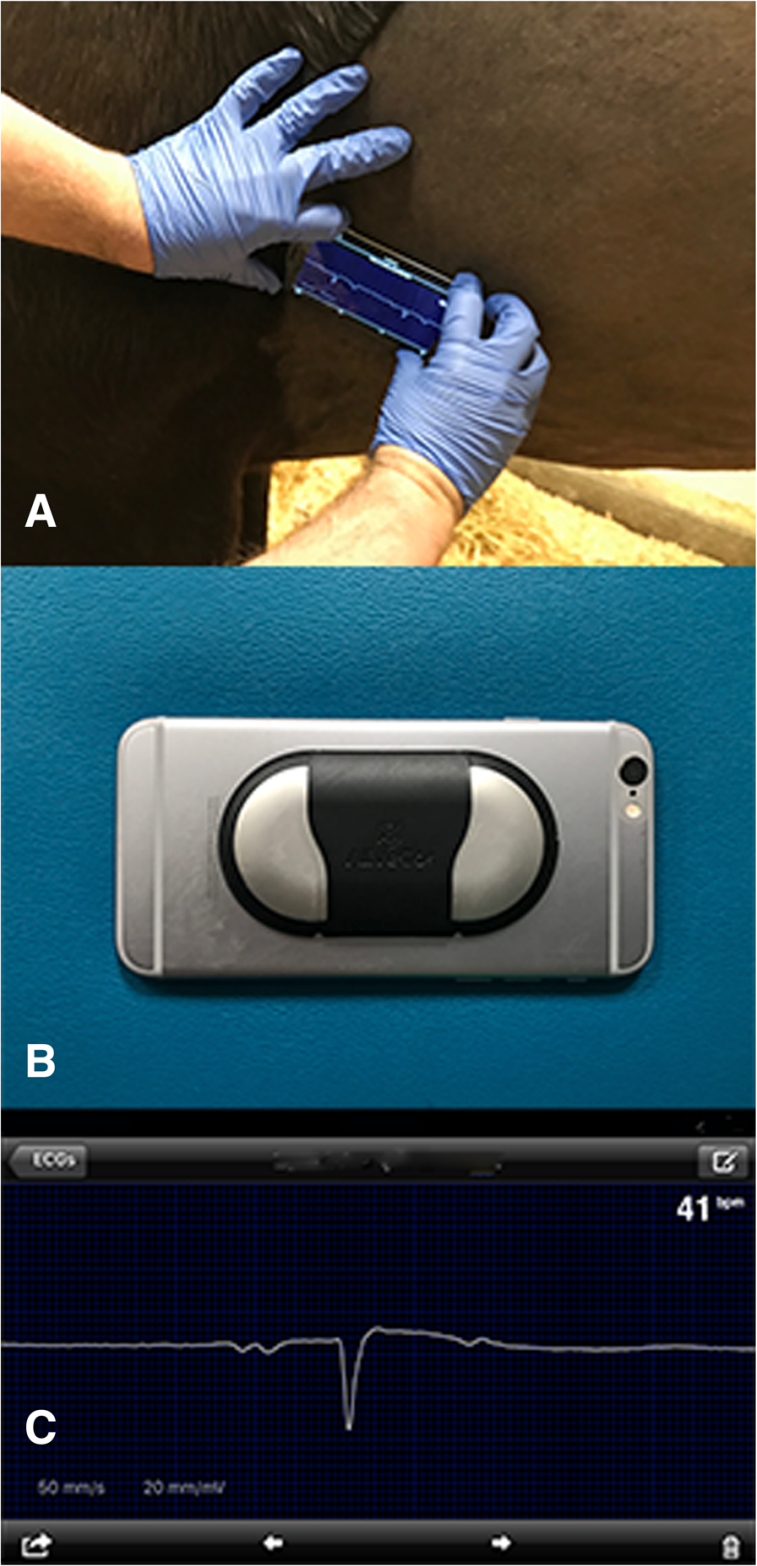
Acquisition of an AliveCor tracing with device placed over the palpated apex beat (**a**), the AliveCor device (**b**), and the ECG tracing obtained at 50 mm/s and 20 mm/mV (**c**)

Electrocardiography studies were performed on all horses 24 to 48 h prior to the race (T0) and within 30–60 min following the race (T1). The ECG recordings were performed using an AliveCor device and application software.^2^ The device was placed over the left cardiac apex (Fig. 1). ECGs were recorded for 3–5 min with a paper speed of 50 mm/s and a calibration of 20 mm equal to 1 mV. All AliveCor tracings were reviewed for quality and discarded if they were poor quality or there was excessive artifact. ECGs were printed, randomized and the observer was blinded. ECGs were evaluated for rhythm and abnormalities of conduction. Heart rate (HR), P wave duration, PR interval, QRS duration, ST segment deviation from baseline, and QT interval were measured offline using manual calipers by a single operator (AS) under the direct supervision of the attending cardiologist (JS). QT intervals were corrected for HR using the Bazett’s formula and reported as QTc [17].

### Echocardiography

All horses were scanned standing and unsedated at 2 time points (T0 and T1) as described [18]. Standard 2-dimensional and M-mode echocardiography from the right side and left sides of the thorax were performed using a the Acuson P300 portable echocardiogram^3^ equipped with a nominal 3 MHz frequency transducer [18]. All measurements were performed blinded in random order, by one board-certified cardiologist (JS), using an offline work station^4^ as previously described [18, 19]. All measurements were the mean for 3 consecutive cardiac cycles. The following dimensions were measured from the M-mode recordings of the left ventricle, imaged from a right parasternal short axis view at the level of the *chordae tendineae*: the interventricular septum in end-diastole (IVSd), the left ventricular freewall at end-diastole (LVFWd), the left ventricular diameter at end-diastole (LVIDd), and at peak-systole (LVIDs). Left-ventricular systolic function was assessed by calculating left ventricular fractional shortening (FS) in the following way: FS% = (LVIDd-LVIDs)/LVIDd × 100%. The maximal left atrial diameter in systole was measured from the right (LADsax) left hemithorax (LADlax). Color Doppler examination of the mitral, tricuspid, and aortic valves were recorded and scored for presence and severity of regurgitation by the subjective color Doppler surface area method [20].

### Blood samples

Twelve milliliters of blood were obtained by jugular vein puncture at time points T0, T1, 4 h after the race (T4) and 24 h post-race (T24). The blood samples were placed into a heparinized blood collection tube and tested within 30 min of collection. Samples were analyzed with an i-STAT point of care analyzer.^5^ Detection of cardiac troponin I (cTnI) with this point-of-care analyzer is based on a 2-site ELISA, which uses 2 independent antibodies, a caprine polyclonal and a murine monoclonal antibody, that recognize independent epitopes of human cTnI, with a lower limit of detection of 0.01 ng/ml [Kraus, 2010 #789]. The analytic sensitivity, based on the manufacturer insert is 0.02 ng/ml and a reportable range of 0.0–50 ng/ml, with any value above this range reported as >50 ng/ml. Values ≤0.02 ng/ml cannot be discriminated; however, the analyzer provides a specific point estimate of either 0.00, 0.01 or 0.02 mg/l. This analyzer has been previously reported to have good agreement with a similar equine bench-top immunoassay [21]. At all time points an i-STAT EC8+ cartridge^5^ was also used to obtain the following values: pH, pCO_2_, bicarbonate (HCO_3_), sodium (Na), potassium (K), ionized calcium (iCa), glucose (Gluc), and hematocrit (Hct%).

### Statistical analysis

Statistical analysis was performed with commercially available software Prism 7.0 and SPSS 22.0.^6^ The difference between echocardiographic measures and electrocardiographic measures pre- and post-race were compared by either a paired t-test if both data sets were normally distributed or by Wilcoxon matched-pairs signed rank test if one or both failed D’Agostino & Pearson normality testing. A value of *P* < 0.05 was considered significant. One-way repeated measures ANOVA with Greenhouse-Geisser correction followed by Tukey’s multiple comparisons test with individual variances computed for each comparison was performed for blood values at all time points if they were normally distributed. Repeated cTnI and electrolyte measures were compared using a Friedmans test and Dunn’s multiple comparisons test if they were not normally distributed. Mean +/− standard deviation is reported for all normally distributed data and median and interquartile range reported for non-normally distributed data. Where significant differences are identified between time points, the mean or median difference is reported followed by the 95% confidence interval to demonstrate the effect size. Reference ranges for ECG and echocardiographic data were computed using a non-parametric approach (ie. without any distributional assumptions) using percentiles 0.025 and 0.975 to report 95% reference range [22]. Computations were performed using the reference interval package in R language version 3.3.1.^7^ Reference ranges for cTnI, electrolytes, and other biochemical date were computed using the Robust method and reported as 90% confidence intervals in accordance with the American Society for Veterinary Clinical Pathology Guidelines [23, 24].

## Results

Thirty-eight Thoroughbred race horses were evaluated from 23 races, with 1–4 horses evaluated per race. Eight horses were excluded because they were claimed and were unavailable for post-race evaluation. Of the 30 horses evaluated pre- and post-race, there were 13 fillies (<5 yrs. of age), 5 mares (>5 yrs. of age), 8 geldings, and 4 colts (<5 yrs. of age). Ages ranged from 3 to 6 years with a mean of 3.8 (+/− 0.9) years. Horses placed 1st through 12th in their races. Length of race ranged from 5.5 furlongs [1106 m] to 9 furlongs [1810 m] with the most common distance being 6.5 furlongs [1308 m] raced by 12 horses. Eighteen horses raced on a dirt track while the remaining 12 horses raced on turf. Furosemide doses were available for 27 horses; 23 horses received 250 mg. The remaining 4 horses received 150 mg, 350 mg, 400 mg, and 500 mg respectively. For the 3 horses for which specific doses were not available the trainer reported a dose range of 150-350 mg given. Given the lack of variability in this data, no statistical testing was performed on the impact of furosemide dose administered.

### Electrocardiogram

ECGs were available for all 30 horses at T0 and T1. All AliveCor tracings were readable and clear. No dysrhythmias were recorded. Reference ranges for pre- and post-race values are listed in Table 1. Heart rate was significantly increased at the post-race time point with a median HR of 39 bpm at T0 and 86 bpm at T1 yielding a median difference of 39 bpm, 95% CI [31, 58], (*P* < 0.0001). The QT interval was shorter post-race with an increase in HR. Once corrected for HR mean QTc was significantly shorter at T1 when compared to T0 with a mean difference of -91 msec, 95% CI [−108, −72], (*P* < 0.0001). PR interval also decreased post-race with a mean difference of -85 msec, 95% CI [−105, −66], (*P* < 0.001). There was no significant change in ST segment deviation between T1 and T0 with a median difference of 0.3 mV, 95% CI [−0.2, −0.5], (P 0.11).

**Table 1:** Selected ECG and echocardiogram parameters recorded prior to race and within 30–60 min after the race start time are listed

	Pre-race (T0)	RI (pre-race)	Post-race (T1)	RI (post-race)	*P* value
ECG					
PR interval (sec)	0.279 (+/−0.049)	0.211–0.381	0.193 (+/−0.035)	0.146–0.259	**<0.0001**
QRS duration (sec)	0.098 (+/−0.013)	0.075–0.118	0.093 (+/−0.012)	0.073–0.113	.0789
QT interval (sec)	0.445 (+/− 0.053)	0.355–0.567	0.353 (+/− 0.047)	0.271–0.436	**<0.0001**
QTc interval (sec)	0.507 (+/− 0.051)	0.438–0.591	0.417 (+/− 0.024)	0.371–0.454	**<0.0001**
ST segment (mV)	1.3 (0.6,2.3)	0.2–2.9	1.8 (+/−1.0)	0.4–3.6	0.1115
HR (bpm)	39 (32,45)	26–63	86 (69,100)	58–126	**<0.0001**
Echocardiogram					
LAD Sax (cm)	10.9 (+/−0.7)	9.8–12.2	10.1 (+/−0.9)	8.4–11.4	**<0.0001**
LAD Lax (cm)	13.0 (+/−0.7)	11.8–14.7	11.8 (+/− 1.1)	10.1–13.5	**<0.0001**
Ao (cm)	7.5 (+/−0.6)	6.6–8.6	7.3 (+/−0.5)	7.4–8.4	0.1547
IVSd (cm)	3.3 (+/−0.4)	2.7–4.0	3.5 (+/−0.4)	2.8–4.2	**0.0117**
LVIDd (cm)	12.1 (+/−1.0)	10.5–13.7	10.9 (+/−1.2)	8.5–13.0	**<0.0001**
LVIDs (cm)	6.9 (+/− 1.1)	5.0–8.7	5.5 (+/−1.2)	3.3–7.4	**<0.0001**
LVFWd (cm)	2.8 (+/−0.3)	2.2–3.4	2.8 (+/−0.5)	2.2–3.7	0.7321
FS (%)	42.2 (+/−7.6)	30.8–58.0	50.0 (+/−8.2)	38.4–67.1	**<0.0001**

Pre- (T0) and post-race values (T1) were compared and the *P* value is reported. The *P* value is bolded when a significant difference between T0 and T1 was present. The QT interval was corrected for HR (QTc) using Bazett’s formula. Normally distributed data is reported in mean +/− standard deviation. Non-parametric data is reported in median with interquartile range in parenthesis. Reported with reference intervals (RI) computed using a non-parametric approach (ie. without any distributional assumptions) using percentiles 0.025 and 0.975 to report 95% reference range

### Echocardiogram

An increase in number of horses with valvular regurgitation was noted across all valves interrogated on echocardiogram after the race (Table 2). Prior to the race 15 horses (50.0%) had no identifiable aortic regurgitation (AR). Following the race only 4 horses had no AR, 12 had trace, and 14 had mild AR. Tricuspid regurgitation (TR) was noted to be absent in 12 horses (40.4%), trace in 13 (43.3%), and mild in 5 horses (16.7%) prior to racing. On Doppler interrogation after the race TR was noted to be mild in 16 horses (53.3%) and moderate in 8 horses (26.7%). No mitral regurgitation (MR) was appreciated in any horses on echocardiogram prior to racing. Mild MR (*n* = 3) and trace MR (*n* = 5) were noted in 8 horses post-race.

**Table 2:** Echocardiographic determination of valvular regurgitation at pre- and post-race time points in 30 Thoroughbred horses

	Pre-Race (T0)	Post-Race (T1)
AoV Regurgitation	Number of Horses (%)	Number of Horses (%)
None	15 (50.0%)	4 (13.3%)
Trace	11 (36.6%)	12 (40.0%)
Mild	4 (13.3%)	14 (46.6%)
Moderate	0	0
TV Regurgitation		
None	12 (40.4%)	4 (13.3%)
Trace	13 (43.3%)	2 (6.7%)
Mild	5 (16.7%)	16 (53.3%)
Moderate	0	8 (26.7%)
MV Regurgitation		
None	30 (100%)	22 (73.3%)
Trace	0	5 (16.7%)
Mild	0	3 (10.0%)
Moderate	0	0

Left atrial dimension and left ventricular internal dimensions decreased significantly with mean differences of −1.2 cm, 95% CI [−1.6, −0.8] and −1.2 cm, 95% CI [−1.5, −0.9] respectively following the race (*P* < 0.0001; Table 1). Fractional shortening % significantly increased after the race when compared to pre-race values with a mean difference of 7.9%, 95% CI [4.8, 10.9], (*P* < 0.0001).

### Blood analysis

**Fig. 2:**
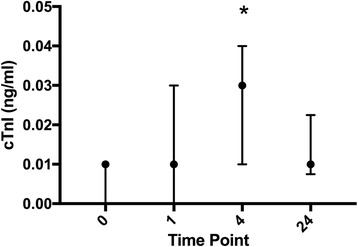
Graphical representation of serum cTnI levels for each horse over time which demonstrates the delayed release pattern peaking at T4 and returning to baseline by T24. Time points are labeled as T0, T1, T4, and T24 which correspond to measurements obtained before the race, 30–60 min after the race, 4 h post-race, and 24 h post-race respectively. Circles represent the median with lines indicating the interquartile range. Significance (*P* < 0.05) is denoted by *

Blood gas values at all time points and reference ranges are noted in Table 3. Pair-wise comparison of cTnI was significantly different between T0 and T4 with a median difference of 0.02 ng/ml, 95% CI [0.01–0.03], (*P* < 0.0001, Fig. 2). Hematocrit significantly increased between T0 and T1 with a mean difference of 10.2%, 95% CI [7.2, 13.3], (*P* < 0.0001), but returned to T0 values by 24 h post-race. Sodium significantly decreased between T0 and T1 with a mean difference of −2.6 mmol/L, 95% CI [−3.8, −1.4], (*P* < 0.0001). Bicarbonate (HCO3) significantly decreased between T0 and T1 with a mean difference of −13.6 mmol/L, 95% CI [−15.6, −11.6], (*P* < 0.0001). Ionized calcium (iCa) significantly decreased between T0 and T1 with a mean difference of −0.30 mmol/L, 95% CI [−0.33, −0.26], (*P* < 0.0001).

**Table 3:** Bloodwork performed via iSTAT cartridges and sampled across all time points (T0 = pre-race, T1 = post-race, T4 = 4 h post-race, and T24 = 24 h post-race), in 30 Thoroughbred race horses

Blood Parameters	T0	T0 RI	T1	T1 RI	T4	T4 RI	T24	T24 RI
cTnI (ng/ml)	0.01 (0.0,0.01)	<0.01	0.01 (0.0,0.03)	0.0–0.05	0.03 (+/−0.02)^a^,+	0.0–0.07	0.01 (0.0,0.02)	0.0–0.05
pH	7.47 (+/−0.03)	7.41–7.52	7.33 (+/−0.11)^a^	7.17–7.56	7.48 (+/−0.05)+	7.40–7.56	7.46 (7.44,7.49)+	7.36–7.57
HCO3 (mmol/L)	29.2 (+/−1.6)	26.4–32.0	15.6 (+/−5.4)^a^	6.0–25.4	28.7 (27.1,30.3)+	24.3–33.3	29.9 (+/−1.5)+	27.3–32.4
pCO2 (mmHg)	40.2 (+/−3.3)	34.4–45.8	27.9 (+/−4.3)^a^	20.5–35.5	37.9 (+/−6.0)+	28.4–49.3	41.5 (40.1–44.7)+	32.3–51.6
Na + (mmol/L)	138 (+/−1.2)	136–140	135 (+/−2.8)^a^	131–141	136 (+/−1.7)+	133–139	139 (+/−1.3)+,^b^	136–140
K+ (mmol/L)	3.7 (+/−0.4)	3.0–4.4	3.5 (+/−0.3)^a^	3.0–4.1	3.5 (3.1,3.7)	2.1–4.9	3.6 (3.4,3.8)	2.8–4.4
iCa++ (mmol/L)	1.63 (+/−0.06)	1.52–1.73	1.34 (+/−0.06)^a^	1.23–1.45	1.56 (+/−0.07)^a^,+	1.44–1.68	1.59 (+/−0.05)+	1.51–1.67
Glucose (mg/dL)	102 (92,108)	78–122	161 (145,195)^a^	94–229	107 (91,127)+	52–159	110 (+/−13)+	85–131
Hct (%)	41.6 (+/−5.7)	31.9–51.7	51.8 (+/−6.8)^a^	40.2–63.8	45.0 (+/−5.7)^a^	34.4–54.1	40.5 (33,43)+,^b^	23.3–57.3

Normally distributed data is reported in mean +/− standard deviation. Non-parametric data is reported in median with interquartile range in parenthesis. Reported with reference intervals (RI) calculated using the Robust method reported as 90% confidence interval in accordance with American Society for Veterinary Clinical Pathology guidelines. All values were compared to each other. Values that are significantly different (adjusted *P* < 0.05) than all other time points are marked as follows: ^a^denotes significance when compared to T0, + denotes significance when compared to T1, ^b^denotes significance when compared to T4

**Fig. 3:**
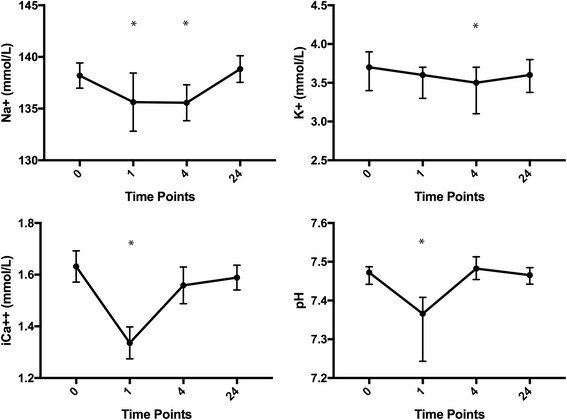
Graphical representation of biochemical values at Time Points T0, T1, T4, and T24 which correspond to measurements obtained before the race, 30–60 min after the race, 4 h post-race, and 24 h post-race respectively. Significant differences relative to T0 are labeled *

Sodium was significantly decreased at T4 with a mean difference of −2.6 mmol/L, 95% CI [−3.4, −1.9], (*P* < 0.0001). Potassium was significantly decreased at T4 with a median difference of −0.4 mmol/L, 95% CI [−0.5, −0.1], (*P* = 0.0307). Both sodium and potassium returned to baseline by T24. Serum pH decreased during the time immediately following the race (T1) with a mean difference of −0.14, 95% CI [−0.18, −0.10], (*P* < 0.0001), but returned to baseline value by T4. Graphical representations of biochemical data are presented in Fig. 3.

No single horse was a consistent outlier across the parameters measured on ECG, echocardiogram, or blood values obtained.

## Discussion

This study describes normal pre- and post-race values for selected ECG, echocardiogram, and blood parameters in competing Thoroughbred racehorses. This study is unique because it represents acquisition and assessment of these values in competitive racing horses and not in the laboratory or after exercise on a treadmill. No consistent outliers were appreciated and there was no suggestion of EICF at the time points studied, based on the measures employed. There was also no incidence of sudden death observed in this study. This finding does not preclude the development of either condition in the future or with subsequent exercise or evaluations. It is important to remember that all horses of this study were trained and actively racing during the season, thus T0 evaluations are not representative of the general non-racing Thoroughbred population.

Electrocardiograms were easily and effectively collected with the AliveCor device. The AliveCor is smartphone-based handheld ECG that delivered readable and interpretable data without the necessity of a cumbersome machine and electrode clips. AliveCor has been validated for rhythm evaluation in humans, dogs, cats, and water buffalo calves [25–28]. Measurement of QT and QTc in using the AliveCor device has reported to have good agreement with 12-lead ECG in humans [29]. This is a viable option for rhythm and QT assessment immediately after exercise on the race track or in the clinic. There was no gold-standard that this device was compared to as this study did not specially aim to validate this method. Further investigation is needed to determine if the values created in this study can be applied to standard base-apex lead measures although given the results from the human and canine studies with the AliveCor device it is likely similar.

Racing ameliorates vagal tone that is dominant at rest [30]. Increased HR recorded at T1 relative to is likely due to increased sympathetic tone after exercise and decreased vagal influence [16]. This also explains the decrease in PR interval recorded after racing (T1) because sympathetic tone can increase conduction velocity through the atrial myocardium.

Alterations in QT interval can place patients at increased risk of sudden death by extending the vulnerable period for inciting severe cardiac arrhythmias or ventricular fibrillation [16]. No dysrhythmias or prolongation in QT or QTc were noted in this study. Sudden cardiac death is of increasing concern post-race and theorized to be due to arrhythmia [9, 10]. However, the low prevalence of sudden death in the racehorse population may have rendered this study underpowered to identify it.^8^ Alternatively, previous reports have noted that vagal rebound is a more important factor in post-race dysrhythmias [9]. Thus the sampling time-point of 30–60 min post-race may have been too early to identify recovery associated dysrhythmias. Additionally, some studies have indicated that prolonged ECG monitoring is necessary to identify the true frequency of cardiac arrhythmias in equine athletes. This may implicate that the short duration ECG used in our study is an underestimate of cardiac dysrhythmias in this population [4, 31]. While this represents a limitation, the use of a 3-5 min duration ECG remains a clinically accessible option for racehorses on the track, while ambulatory ECG monitoring is generally unavailable in this setting.

ST segment alterations suggest changes to cardiac repolarization. The cause of ST segment elevation or depression has not been specifically evaluated in the horse. However, across many other species it serves as an indicator of left ventricular ischemia or hypoxemia [32–34]. The lack of ST segment alteration in this study may be due to the AliveCor’s inability to measure minor electrophysiologic alterations or may indicate that our population did not experience this. Further evaluation comparing AliveCor to standard base apex lead measurements in horse is needed.

The echocardiographic changes in LA and LV dimensions are consistent with what has been previously reported in endurance horses [6]. The noted decrease in LA and LV size may be secondary reduction in preload or due to an increase in HR and thus more forward flow and less diastolic filling. Decrease in these chamber sizes could also be secondary to furosemide administration and the resultant volume reduction.

The increase in FS% may be secondary to sympathetic activation and its positive inotropic effect. Alternatively increase in HR may have contributed to an increased in measured systolic function as a result of the Bowditch effect. The impact of alterations in LV volume secondary to furosemide administration may also impact this observation. Previous studies have noted reported variable valvular regurgitation in athletic horses that has not been associated with deleterious effects on athletic performance [35]. A post-exercise increase in valvular regurgitation was noted in our study. Progressive regurgitation may be in-part due to an increase in systolic function post-race and a resultant increase in the velocity of regurgitation.

Serum cTnI was no different at all time points except T4 (Fig. 2). Cardiac troponin has a delayed release pattern in mice, humans, dogs, and horses [36, 37]. Previous studies in equine athletes did not note a significant elevation when sampled 1–2 h following a race [37]. Our data supported this finding as there was not significant change in cTnI when sampled shortly after the race (T1). There was however an increase in cTnI at T4 which is consistent with troponin’s known delayed release pattern and clearance curves already established [36]. In equine medicine it is assumed but not proven that the magnitude of cardiac damage is correlated with the magnitude of cTnI release. If this is the case, the horses in this study had minor cTnI change and all returned to baseline values by the 24 h time point. This would indicate that a transient release of troponin from the cytosol occurred and permanent or more long-lasting damage to the myocyte did not occur. Theories about transient myocardial hypoxemia have been offered to explain mild increase in cTnI following races [11, 37–39]. Our study would support this notion and suggest that identification of ischemic changes secondary to racing would be best evaluated by measurement of cTnI at 4 h and 24 h post-race, rather than immediately after the race. Alternatively, an undetectable amount of troponin may be released earlier than measured, however this value may not reach detectable levels until at least 4 h after the race. Further investigation using a high- or ultra-sensitivity assay may be considered to evaluate this possibility.

As expected with anaerobic exertion, pH decreased after the race likely due to lactic acidosis from anaerobic metabolism [40]. This is a tightly regulated value and was observed to return to baseline by T4. Partial pressure of CO2 decreased immediately after the race consistent with previous reports [40]. This is hypothesized to be due to either post-race hyperventilation in an effort to recover from this extreme exercise and/or respiratory compensation of metabolic acidosis from anaerobic metabolism and increased lactic acid [40].

While there was a statically significant alteration in Na + values in our study at T1 and T4, however given the overlap in the ranges of these values the clinical significance of this finding is unclear. Previous studies have identified hyperkalemia immediately after exercise likely secondary to transmembrane fluxes from exercising muscles [41]. In contrast, in our study the K values measured were not significantly decreased immediately following the race (at T1) and became significantly decreased at 4 h post-race. The lack of change at T1 could be due the slight delay in sampling time or due to a balance between loss and increase. For instance, loss secondary to furosemide administration together with an increase due to efflux from the intracellular space during extreme exercise may have resulted in a net negligible change in this value. The reduction in K at T4 is most likely due ion loss secondary to furosemide administration [42]. The glucose increase noted is thought to be due to increased sympathetic stimulation [43].

There was a notable increase in Hct consistent with previous studies which could be due to splenic contraction following excitement and exercise known to occur in horses [5, 44]. Increase in Hct could also be due to plasma volume depletion from furosemide administration and post-race dehydration [42]. Hematocrit returned to pre-race values by 4 h after the race. This supports the notion that the increase is transient with a rapid recovery.

There are a number of limitations for this study inherent in performing a field based study. A principal limitation is that all horses were treated with furosemide between T0 and T1. Separating if the observed effects of this study are secondary to exercise, furosemide or a combination of both is not possible using the present study design and may represent a future aim for a study to be completed in horses where furosemide may be withheld. The trainers whose horses were enrolled in this study were unwilling to alter their racing practices to exclude furosemide.

Sample size is a common limitation in veterinary studies and this study is no exception. The access to high-level competing horses immediately post-race is challenging and the enrollment of 38 horses and study completion of 30 horses generated a significant dataset for generation of reference intervals. It is possible that with a larger sampling of horses, more outliers could have been identified and more narrow reference intervals created.

The working nature of these horses significantly limited the extent of echocardiographic and electrocardiographic evaluations that could be performed, as time for evaluation was brief. The authors selected a small number of study variables that may be readily accessible for screening horses in this environment. More robust systolic and diastolic function assessments may have elucidated subtle alterations in function post-race however many diastolic assessments have proven to be technically difficult and unreliable post-exercise [19]. For this reason, horses with EICF could have been overlooked. Fractional shortening, like many systolic function assessments, is both preload and afterload dependent thus the alternations noted to this value may reflect alterations in these factors. The assessment of valvular regurgitation was subjective in nature and thus may be prone to error. Currently there is no accepted gold standard quantitative assessment of valve regurgitation in horses or human athletes and many varieties of assessment rely on subjective qualitative assessment. This assessment was performed by a single investigator to decrease this variability and has been used for evaluation of regurgitation in previous studies [20, 45].

No horses in our study had detectable evidence of EICF and no sudden death events were noted. Identification of horses that demonstrated EICF or sudden death would provide highly valuable information in these conditions. However, the low incidence of EICF or sudden death suggests that designing a study to identify this condition would require massive sample size which was not feasible within the scope of this work. The use of the reference ranges established in this study can however be used to help to early detect horses at high risk of EICF in future races and studies.

Another limitation was in the precise timing that the measurement for ECG and echocardiogram were gathered. Inherent to the nature of racing and the number of animals included, ECGs and echocardiogram evaluations, were performed at a variable time within the first hour post-race. This may have allowed for some cardiovascular recovery and subsequently increased variability in the reference intervals obtained.

Additionally, the ECG measurements were obtained using an AliveCor device which has not been previously validated in horses. In humans, dogs, and cats this device has been used to assess for arrhythmias and ECG wave-form duration but its value in assessment of complex height is limited given the variation that is associated with placement of electrodes on the animal. Complex height was not measured in this study for this reason. It is highly likely that given the nature of ECG duration measure, the values obtained with the AliveCor reflect those that would be obtained with a standard base-apex lead. This however was not a goal of this study and further investigation is needed to confirm this association.

It is also important to note that the reference intervals generated were determined for the iSTAT Chem 8 and cTnI assay and may not necessarily be extrapolated to other devices as there is known variability in cTnI assays [37].

Finally, this study utilized a before and after study design where each horse served as its own control. While this approach is commonly employed and results in greater statistical power with a smaller number of animals, it is subject to a phenomenon called regression to the mean [46]. The impact of possible regression to the mean on this data set cannot be determined.

Further areas of study include the screening of more animals with the protocol established in this study to help identify outliers particularly if there is a suggestion of EICF or decreased athletic performance. Further evaluation in order to correlate the parameters measured in this study with performance outcomes of success on the track is an area of future investigation. Finally following a number of individual horses and their trends over time and subsequent races may help to better identify animals that are at risk of fatigue.

## Conclusion

The reference intervals generated in this study may be used to identify racing Thoroughbred horses with inappropriate electrophysiological, biochemical or echocardiographic responses to racing.

## Abbreviations

AR: Aortic regurgitation
cTnI: Cardiac troponin I
ECG: Electrocardiogram
EICF: Exercise induced cardiac fatigue
FS: Shortening fraction
Gluc: Glucose
HCO3: Bicarbonate
Hct: Hematocrit
HR: Heart rate
iCa: Ionized calcium
IVSd: Interventricular septum in end-diastole
K: Potassium
LADlax: Left atrial dimension long-axis
LADsax: Left atrial dimension short-axis
LVFWd: The left ventricular freewall at end-diastole
LVIDd: The left ventricular diameter at end-diastole
LVIDs: The left ventricular diameter at peak-systole
MR: Mitral regurgitation
Na: Sodium
T0: Pre-race time point
T1: 30–60 min post-race time point
T24: 24-h post-race time point
T4: Four-hours post-race time point
TR: Tricuspid regurgitation

## Footnotes

1 California Horse Racing Board (2013) 43rd Annual Report. www.chrb.ca.gov (Publications). Sacramento, CA.

2 AliveCor, Inc. San Francisco, CA 94108, USA.

3 Siemens Medical Solutions USA, Inc. Malvern, PA 19355, USA.

4 syngo Dynamics, Siemens Medical Solutions, Malvern, PA.

5 Abbott Point of Care Inc., Princeton, NJ 08540, USA.

6 GraphPad Software, Inc. La Jolla, CA 92037, USA.

7 R Core Team. 2016. R: A Language and Environment for Statistical Computing. Utilizing R Reference Interval Package (referenceInterval” package in R (https://cran.r-project.org/web/packages/referenceIntervals/referenceIntervals.pdf) R Foundation for Statistical Computing. Vienna, Austria.

8 California Racing Board (2014) Baffert Sudden Death Report. www.chrb.ca.gov/veterinary_reports/baffert_sudden_death_report_final_1121.pdf
